# Engineered Two-Dimensional Nanostructures as SERS Substrates for Biomolecule Sensing: A Review

**DOI:** 10.3390/bios13010102

**Published:** 2023-01-06

**Authors:** K. A. Esther Jebakumari, N. K. Murugasenapathi, Tamilarasan Palanisamy

**Affiliations:** 1 Electrodics and Electrocatalysis Division (EEC), CSIR—Central Electrochemical Research Institute (CECRI), Karaikudi 630003, Tamil Nadu, India; 2 Academy of Scientific and Innovative Research (AcSIR), Ghaziabad 201002, Uttar Pradesh, India

**Keywords:** surface-enhanced Raman spectroscopy, chemical enhancement, two-dimensional nanostructures, engineered substrates, biomolecule sensor

## Abstract

Two-dimensional nanostructures (2DNS) attract tremendous interest and have emerged as potential materials for a variety of applications, including biomolecule sensing, due to their high surface-to-volume ratio, tuneable optical and electronic properties. Advancements in the engineering of 2DNS and associated technologies have opened up new opportunities. Surface-enhanced Raman scattering (SERS) is a rapid, highly sensitive, non-destructive analytical technique with exceptional signal amplification potential. Several structurally and chemically engineered 2DNS with added advantages (e.g., π–π* interaction), over plasmonic SERS substrates, have been developed specifically towards biomolecule sensing in a complex matrix, such as biological fluids. This review focuses on the recent developments of 2DNS-SERS substrates for biomolecule sensor applications. The recent advancements in engineered 2DNS, particularly for SERS substrates, have been systematically surveyed. In SERS substrates, 2DNS are used as either a standalone signal enhancer or as support for the dispersion of plasmonic nanostructures. The current challenges and future opportunities in this synergetic combination have also been discussed. Given the prospects in the design and preparation of newer 2DNS, this review can give a critical view on the current status, challenges and opportunities to extrapolate their applications in biomolecule detection.

## 1. Introduction

Biomolecule detection and quantification have become increasingly important in recent years, due to advancements in clinical diagnosis, which requires newer technologies for rapid and accurate detection of molecules at ultratrace concentrations. Since the historic development of enzymatic electrodes by Clark and Lyons in 1962 [1], there has been a quest among researchers for advanced sensing technologies and this has resulted in the development of more sophisticated and trustworthy sensors [2,3,4]. Though several techniques have emerged, Raman spectroscopy has sparked the most interest in biomolecule sensing due to its exceptional sensitivity rendered by the large signal amplification, chemical specificity, rapid recognition and non-destructive nature. Raman spectroscopy identifies the characteristic molecular vibrations and provides the fingerprints of the molecules with minimal to no sample preparation. However, the weak signal, due to low scattering probability (typically 10^−4^–10^−6^), was a bottleneck when deploying this versatile technique in the detection of ultratrace target molecules, until the discovery of Surface-enhanced Raman scattering (SERS) by Fleischmann et al., in 1974 [5]. The observation of an enhanced Raman signal of pyridine on roughed Ag electrodes eventually helped Raman spectroscopy to extend its applications up to the detection of a single molecule [6,7].

The electromagnetic (EM) and chemical (CM) mechanisms are the two important phenomena behind the Raman signal enhancement, proposed later by Van Duyne and Creighton groups, independently, in 1977 [8,9]. The EM enhancement originates from the excitation of surface plasmon on nanoscale plasmonic surfaces, mainly noble metal nanoparticles (Au and Ag), which contributes dominantly (10^3^ to 10^8^ times) to the SERS enhancement. It is mainly determined by the material morphology, dielectric constant of the medium and the localization of surface plasmon resonance (LSPR) and their coupling [10,11,12,13].

The EM mechanism does not explain about the SERS enhancement with non-plasmonic substrates, e.g., oxides, nitrides, chalcogenides, etc. This can be well understood by the formation of charge-transfer complex, and thus new electronic states, of chemisorbed molecules with the substrates [14]. The CM enhancement is mainly determined by the Fermi level of the substrates and the molecules. The contribution from CM is relatively weaker (up to 10^3^ times) than that of the EM effect. However, CM has comprehensive advantages over EM, including cost-effectiveness, surface uniformity, signal reproducibility, muted photo-bleaching and blinking effects. Further information about the mechanism of SERS can be found in the excellent book by Eric and Pablo [15]. Considerable advancements in understanding charge-transfer complex formation and designing structurally, chemically engineered substrates have been made in the past two decades for the detection of multi-fold trace chemicals and biomolecules, which includes RNA analysis from plant tissues and multiplexed detection at a single-cell level [16,17,18].

**Figure 1:**
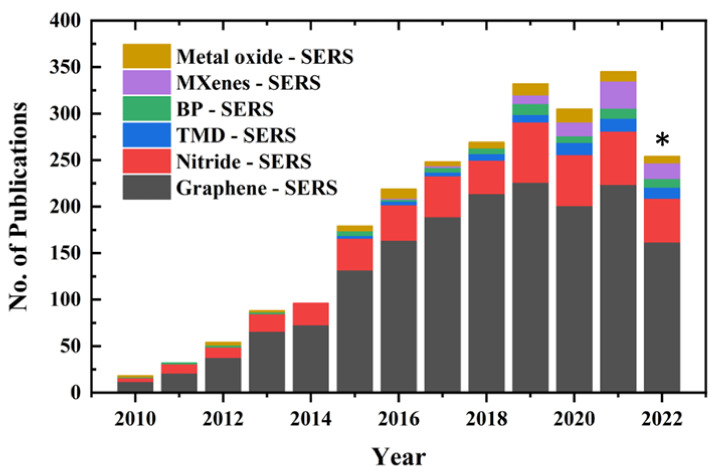
An indicative trend in number of publications on 2DNS-based SERS in the last decade. The search with the keyword “SERS” was refined with “Graphene, Nitrides, Transition-metal dichalcogenides, Black Phosphorus, MXenes and Metal Oxides”, separately. * Data obtained from ISI Web of Science on 27 November 2022.

The discovery of graphene by Novoselov and Geim in 2004 [19] opened a new era in the material sciences, which leads to the further development of various two-dimensional nanostructures (2DNS), including transition-metal dichalcogenides (TMDs), oxides, graphitic carbon nitride (g-C_3_N_4_), hexagonal boron nitride (h-BN), black phosphorus (BP) and 2D transition-metal carbide or nitride (MXenes) [20,21,22]. Recently, nanosheets of metal organic framework (MOF) and covalent organic framework (COF) have also joined the fascinating world of two-dimensional nanostructures. Apart from easy synthesis, these 2DNS and their nanocomposites have several advantages in SERS because of their unique physical and chemical properties, such as high uniformity with large specific surface areas, better chemical stability, excellent mechanical and optical properties with fluorescence quenching capability, π-π* interaction with biomolecules and good biocompatibility [23,24,25]. A detailed review of the advancement of 2DNS-based SERS substrates and their applications is found in these excellent review articles [23,25,26,27,28,29]. As seen in Figure 1, interest in 2DNS-based SERS has grown rapidly in the last decade.

Although a few reviews have covered the biosensing applications of these 2DNS-SERS in part [30,31], we found no dedicated review on this topic, to the best of our knowledge. In this review, we comprehensively summarize the recent development in 2DNS-SERS, specifically for biomolecule sensing, under two major topics: (i) 2DNS as a SERS substrate and (ii) 2DNS as support for plasmonic SERS substrate. The reports are summarized based on the type of 2DNS to incline the discussions towards the materials aspect. This review may serve as a useful reference for researchers working in the fields of material science, Raman spectroscopy and biomolecule sensing.

## 2. 2DNS as SERS Substrates

**Figure 2:**
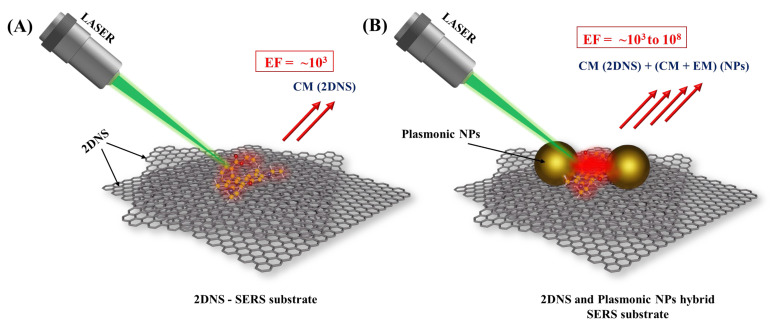
Schematic illustration of (**A**) 2DNS SERS substrate rendering enhancement through CM and (**B**) 2DNS as support for plasmonic NPs SERS substrate that enhances Raman signal by both CM (from 2DNS and NPs) and EM (from NPs).

As mentioned earlier, the Raman signal enhancement by 2DNS is mainly through a charge-transfer mechanism. The electronic structure of the analyte–substrate interface, which is primarily accomplished by the transfer of an electron from the highest occupied molecular orbital (HOMO) to the lowest unoccupied molecular orbital (LUMO), determines the contribution of CM (charge-transfer) to Raman signal amplification. Moreover, the π-interaction facilitates the accumulation of analytes on their surface, which has a significant effect at lower concentrations. On the other hand, 2DNS can anchor the plasmonic nanostructures for better dispersion, i.e., prevention of agglomeration. Here, the SERS enhancement factor (EF), the degree of signal amplification [15], is improved as the essential nano-gaps are created by the well-separated plasmonic nanostructures. Therefore, 2DNS were widely deployed for the later purpose. Figure 2 illustrates the use of 2DNS as a SERS substrate and support for nanostructured plasmonic SERS substrates. Table 1 lists representative examples of various 2DNS employed as SERS substrates and support for plasmonic NPs. This section will review the recent developments in 2DNS SERS substrates.

### 2.1. Graphene SERS (GERS) Substrates

Graphene is a single sheet of sp^2^-bonded carbon atoms in a hexagonal honeycomb lattice. It is the well-known and most explored two-dimensional allotrope of carbon with unusual electronic, optical properties, and high theoretical surface area [67,68]. The free π-electron, rich in graphene, can make π-interaction with other systems and accumulate on its surface. Consequently, the charge-transfer between the graphene substrate and the adsorbed molecules is enhanced to observe the SERS signal augmentation [69]. This phenomenon has been exploited in graphene-enhanced Raman scattering (GERS) for a wide range of applications, including materials development [70], energy [71] and biomedicine [72,73].

### 2.2. Nitrides SERS Substrates

The lone-pair electrons in nitrides have an advantage while using them as SERS substrates. A hexagonal lattice made up of boron and nitrogen atoms makes up the equivalent of graphene, known as hexagonal boron nitride (h-BN). Boron nitride possesses a dipole-coupled Raman amplification mechanism, according to a recent investigation [74]. Highly sensitive, label-free, and non-destructive biomolecule detection is achieved using h-BN nanostructures [75]. However, their wider band gap (~6 eV) requires high excitation energy for a conventional CM signal enhancement, which is not suitable for biological molecules [76].

Carbon nitrides are other important 2DNS for Raman signal amplification. Redemann et al. discovered in 1940 that graphitic carbon nitride (g-C_3_N_4_) possesses a graphite-like van der Waals layered structure [77]. Despite having good physicochemical stability, the poor signal enhancement from pristine g-C_3_N_4_ has hindered its use as an independent SERS substrate for sensing applications. However, chemical and structural (e.g., induced disorders to the heptazine chain) modifications may help improve the enhancement factor.

Few compound nitride thin films have also been reported as SERS substrates due to their resonant plasmonic characteristics. For example, Shaoli et.al. have prepared titanium nitride (TiN), aluminium nitride (AlN) and titanium-aluminium nitride (TiAlN) thin film SERS substrates with 95% higher signal strength compared to bare glass substrate [78]. A highly stable niobium nitride thin film with good uniformity has been prepared by reduction nitridation that enhances the Raman signal of Rh6G by 4 × 10^3^ factor [79].

### 2.3. Black Phosphorous (BP) SERS Substrates

Bulk BP was first synthesized in 1914, however, an atomically thin BP 2DNS is realized just recently [80]. Compared to red and white phosphorus, BP is the most stable form of elemental phosphorus [81]. The 2D zig-zag structure of BP sheets consists of phosphorus atoms with three covalently bonded nearest neighbours, while the sheets are bound together by weak van der Waals forces. These layers can be easily exfoliated into 2D BP nanosheets, since the multi-level quantum chemical calculations indicate an exfoliation energy of −151 meV per atom [82]. These wrinkly sheets of honeycomb lattice have armchair and zigzag forms, as in graphene. The layer-dependent band gap, from 0.3 (bulk) to 2.0 eV, of BP allows the use of a wide range of excitation light in the UV, visible and NIR ranges for SERS analysis [83]. Interestingly, Lin et al. reported an anisotropic SERS substrate using few-layered BP and ReS_2_, which exhibited polarization-dependent signal enhancement [84]. Therefore, BP nanosheets have recently received great attention for a wide range of applications, particularly in biomedicine, photothermal therapy, photodynamic therapy, drug administration, 3D printing, bio-imaging, and theranostics [85,86,87].

### 2.4. MXenes SERS Substrates

Transition metal carbides, nitrides or carbonitrides make a new class of 2D material, known as MXenes. They typically have a layered structure with (*n* + 1) layers of *M* connected by *n* layers of *X* in the pattern *[MX]_n_-M*, where *M* is an early transition metal (such as Sc, Ti, Zr, Hf, V, Nb, Ta, Cr or Mo), and *X* is either carbon or nitrogen. A general formula for these compounds is *M_n+_*_1_*X_n_* (*n* = 1–3) [88,89]. Since its first discovery (Ti_3_C_2_) in 2011, MXenes have attracted immense attention in a variety of applications, including energy, environmental and healthcare sectors. The high electrical conductivity of highly metallic MXenes, having unique electronic and optical properties and intense LSPR effect in the visible or near-infrared range, makes them a promising SERS substrate [58]. Here, both EM and CM contribute to boosting the Raman signal [90]. Their flexibility and hydrophilic nature make functionalization or tagging with Raman reporters, easy.

### 2.5. Transition Metal Dichalcogenide (TMD) SERS Substrates

Compounds with the generalised formula *MX_2_*, where *M* is a transition metal and *X* is a chalcogen, such as S, Se or Te, make up the family of layered materials known as “transition metal dichalcogenides”. Strong intralayer bonding and weak interlayer binding enable the exfoliation of these van der Waals solids into 2D nanosheets [91]. A layer of transition metal sandwiched between two saturated chalcogen layers makes these less reactive 2D TMD layers. The confinement of charge carriers in two dimensions in TMDs dramatically alters their characteristics for a variety of applications [30,92]. These atomically flat sheets enable effective charge transfer between the probe molecules and substrates through weak contacts, such as π–π* interactions, and make them suitable for chemical Raman signal enhancement [93,94]. These TMDs are particularly interesting since they facilitate attachment of probed molecules to induce the CM effect [95].

### 2.6. Metal Oxide SERS Substrates

Most semiconductors exhibit weak SERS signals due to their large band gaps and lack of surface plasmon resonance. Oxygen incorporation in semiconductors increases the Raman enhancement factor as good as 10^5^ times, probably due to the enhanced charge-transfer from the semiconductor band edges to the adsorbed molecules [96]. Metal oxide semiconductors, such as titanium oxide (TiO_2_), tungsten oxide (WoOx) and molybdenum oxide (MoOx), were recently tested as SERS substrates [43]. The surface polarisation effect due to the oxygen defect states boosts the Raman signals in these substrates [97]. For instance, few-layered MoO_3_ nanosheets act as a sensitive SERS substrate, which enhances the Raman signal up to 2.28 × 10^4^ times and makes it capable of detecting 2 × 10^−8^ M of an Rh6G molecule [98]. Similarly, ultrathin, chemical vapour-deposited MoO_2_ nanosheets show enhancement of the Raman signal up to 2.1 × 10^5^ and possess excellent reusability and uniformity [99]. In both cases, it has been found that the EF further increased by decreasing the thickness of the MoO_x_ nanosheets.

### 2.7. 2D MOFs/COFs SERS Substrates

Metal-Organic Frameworks (MOFs) are crystalline porous materials consisting of metal ions or cluster nodes linked by organic ligands such as carboxylate ligands and other negatively charged ligands [100,101]. MOFs show excellent SERS performance that is generally attributed to the charge transfer enhancement mechanism [102]. Several studies have been carried out to deploy MOFs as SERS substrates. For the first time, Yu et al. reported the Raman signal enhancement of Methyl Orange adsorbed on Matériaux Institut Lavoisier (MIL)-type MOFs [103]. Later, several other MOFs, including ZIF-67, Co-TCPP MOFs and Co-MOF-74 were employed directly as SERS substrates, which shows an EF of about 10^6^ for an Rh6G molecule [104]. Covalent Organic Frameworks (COFs) are ordered structures built up from organic building blocks via covalent bonds [105]. The use of COFs as SERS substrate is still in its infancy, while MOFs gained more popularity because of the plasmonic hybrids. Two-dimensional allotropes of these MOFs and COFs are attracting increasing research attention due to their ultrathin morphology, which offers a high surface-to-volume atom ratio [100]. Their high surface area with molecular structure facilitating a π–π* interaction is a critical advantage for their application in SERS substrates.

## 3. 2DNS-Based SERS Biomolecule Sensors

This section reviews the potential of various 2DNS, discussed in Section 2, as SERS substrates for biomolecule detection. The hydrophobic sp^2^ and sp^3^ structures of graphene allow easy functionalization with oxygen-containing functional groups, such as carboxyl, epoxy, hydroxyl and carbonyl groups to make it hydrophilic, known as graphene oxide (GO). The physicochemical properties of GO can be precisely tuned by these oxygen-containing functional groups. Moreover, the hydrophilic nature of GO gives its biocompatibility [106], interaction with hydrophilic moieties [107] and inhibits biofouling [108]. These groups enhance affinity beyond the π-interaction and enhance water diffusibility, which is essential for sensing biomarkers in biological fluids [109].

Similarly, 2D nitrides also have shown good potential towards Raman signal enhancement. Recently, an EF of ∼10^5^ was achieved by fabricating transition-metal nitrates such as tungsten nitride (WN) and tantalum nitride (TaN) chips as SERS substrates [110]. An additional twofold signal enhancement was attained by constructing a nano-cavity structure such as these nitride chips, which would be sufficient for ultratrace biomolecule detection. Similarly, nano-voids formed in BP sheets induce the intrinsic in-plane ferroelectric property and result in the SERS EF as high as ∼10^6^ and LOD as low as ∼10 nM of RhB [49]. The unique physicochemical properties and recent technological advancement to achieve signal enhancement comparable to plasmonic substrates indicate the potential of 2D carbon, nitride and BP nanostructures as a standalone SERS signal enhancer. However, it needs further dedication to engineer the substrates and test them with various biomolecules.

TMDs are the mostly used 2DNS for biomolecule sensing applications. For instance, MoS_2_ nanosheets were used to make a SERS-based immunoassay for the monitoring of carbohydrate antigen 19-9 (CA19-9) with good sensitivity and specificity. Effective molecular enrichment on the large active surface area of MoS_2_ and potential charge-transfer resonances caused by the 532 nm laser resulted in a 10^5^ enhancement factor. Intriguingly, a sandwich immune-complex using MoS_2_ nanoflower and nanosheet demonstrated a LOD for CA19-9 as low as 3.43 × 10^−4^ IU/mL, in addition to a broad linear range from 5 × 10^−4^ to 1 × 10^−2^ IU/mL matching the clinical levels [111].

A few-layered Hafnium ditelluride nanosheet SERS substrate has been developed for detection of uric acid, an important biomarker for gout disease, with a verified LOD of 100 μM [45]. The semi-metallic MoTe_2_ SERS substrate exhibited enhancement depending on the number of layers in the films. Here, the signal enhancement is a result of surface–dipole interaction, the ability of the analyte to become polarized in contact with the surface. Fraser et al. demonstrated SERS-based detection of β-sitosterol on MoTe_2_ films [44].

Another 2DNS having exceptional potential as a SERS substrate for biomolecule sensing is MXenes. They have a SERS enhancement factor as high as that of plasmonic substrates with the added advantage of a 2D structure. MXenes, such as Ti_2_C, Nb_2_C and Ta_2_C, have been developed with Raman signal EF of ∼10^6^ [90,112]. A surprising signal enhancement, as high as 10^12^ times, has been reported using Ti_2_N MXenes on paper, silicon and glass substrates [58]. Recently, MXenes SERS substrates were deployed for the detection of SARS-CoV-2 protein, which could detect at a LOD of 5 nM [112]. This relatively young 2DNS has tremendous untapped potential as a SERS substrate.

Although 2D MOFs/COFs have gained more popularity among researchers in the past decade for a variety of applications, including catalysis, energy storage and gas adsorption, their applications in SERS substrates have only been explored very recently [103]. However, their applications in biomolecule sensing are yet to be explored. A few attempts have been made to use 2D MOFs/COFs as support for a plasmonic SERS substrate, and are discussed in Section 4.6.

## 4. 2DNS as Support for Plasmonic Nanostructure in SERS Biosensors

The high surface area, ease of functionalization and chemical stability of 2DNS make them ideal support for dispersing metallic nanostructures, which are generally prone to agglomerate [113]. Particularly, electrically conductive nanosheets, such as graphene and TMD, were used as a catalyst support in fuel cells, electrolysers, solar cells and batteries [114]. On the nanostructured plasmonic SERS substrates, the signal enhancement is coupled with the field enhancement, which requires LSPR coupling. Creating a nano-gap is crucial for confining/localizing surface plasmon resonance. As recognized widely, functionalized 2DNS can be used as a support for dispersing the plasmonic nanostructures where the functional groups can act as anchoring sites. In addition, these 2DNS can facilitate the accumulation of analytes and the formation of a charge-transfer complex, as discussed in Section 2. This section will focus on 2DNS-supported plasmonic nanostructure as a SERS substrate for biomolecule sensing. A comparison of such 2DNS-supported SERS substrates is given in Table 2.

### 4.1. Graphene-Supported SERS Substrates

**Figure 3:**
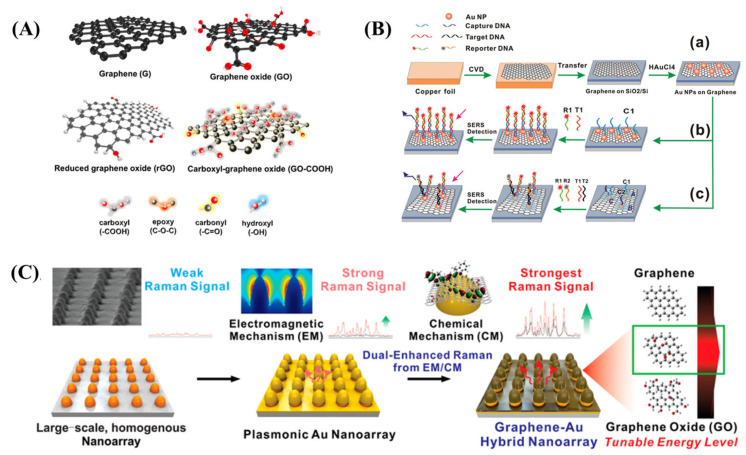
(**A**) Graphene and its derivatives [31], (**B**) Schematic illustration of the fabrication of SERS-active substrate and its application for DNA detection. Step (**a**) shows the graphene growth on copper foil by CVD, Step (**b**,**c**) represents the detection of single target and multiplex detection of two different target DNAs, respectively, by GO-Au hybrid SERS sensor [115]. (**C**) Schematic diagram illustrating graphene-coated AuNPs SERS nanoarray for the characterization of neuronal differentiation [143].

Graphene is recognized to be the most suitable catalyst support for electrocatalytic applications, due to its high theoretical surface area (2629 m^2^/g), electrical conductivity, electrochemical stability and ease of functionalization. The functional groups, such as carboxyl, hydroxyl, amine, mercapto and even structural defects (Figure 3A), on graphene, can act as anchoring sites. Therefore, graphene can be a good support for dispersing plasmonic nanostructures as a SERS substrate. The ease of hybridization of graphene with noble metals and increased molecular adsorption are the rationale behind the prominence of noble metal-decorated graphene SERS substrates [135,136]. It is proven that the Raman signals with graphene-supported Au and Ag nanoparticles are stronger than their constituent counterparts [137].

Functionalized graphene and GO have been used as SERS substrates for biomolecule sensing as well [138]. A sandwich assay of functionalized AuNPs has been developed, where the short-length DNA capture probe-functionalized AuNPs were dispersed on graphene oxide. The reporter complex made up of Raman dye (Cy3)-tagged AuNPs makes an Au–analyte–Au sandwich, which results in a high SERS signal amplification. As a result, the biosensor attained good sensitivity and LOD as low as 10 fM [117]. A similar GO-AuNPs and AuNPs dual platform SERS substrate was developed using uniquely designed Raman tag intercalated short-length probe sequences for the simultaneous and quantitative detection of a meat adulterant and an endangered species [118].

The large signal enhancement by combined electromagnetic and charge-transfer mechanisms in 2DNS-supported plasmonic SERS substrates can be used for label-free detection. A label-free SERS probe was developed using GO and popcorn-shaped AuNPs for the detection of HIV DNA at the femtomolar level [116]. Here, the dispersion of the nanostructure, apart from the unique morphology of AuNPs, plays a crucial role. Similarly, graphene-supported label-free sensors for cancer cell profiling [139] and identification of β-amyloid for Alzheimer’s disease diagnosis have also been demonstrated [140].

As graphene functionalization is robust, attaching the biorecognition probe to graphene would be a rational approach. He et al. developed a unique sandwich-type assay using AuNPs dispersed on graphene by chemical vapour deposition (CVD) [115]. Here, short-length sequences were used for capturing and reporting. The capture sequence was attached to AuNPs while the reporter sequence was tagged with Cy3 and tetramethylrhodamine. The traditional “sandwich” shape is a result of the length compensation, as given in Figure 3B. This specifically designed multiplex SERS biosensor had an LOD of 10 pM, allowing it to simultaneously detect the hepatitis A virus Vall7 polyprotein gene (HVA) and the hepatitis B virus surface antigen gene (HVB) with exceptional sensitivity [115]. Huang and colleagues have reported the detection of prostate-specific antigen (PSA) down to 0.23 pg mL using a SERS immunosensor [121].

Copper and silver nanostructure-based SERS substrates often have low physical stability caused by oxidation, which has a significant impact on their sensitivity and efficiency. To mitigate this limitation, SERS-active plasmonic nanostructures are often covered with a stable shield made of inert substances, such as metal oxides and carbon compounds [141]. Due to the exceptional chemical and thermal stability, graphene and GO have been used as a shell. The attenuation from these shells is minimal since they are 98% (per layer) transparent in visible regions [142]. In addition, the accumulation of analyte and efficient charge-transfer complex formation can also improve the signal strength.

Recently, a graphene-coated homogeneous Au nanoarray has been developed to take advantage of EM by Au nanoarrays and CM by graphene (Figure 3C). The substrate was used for the neuronal differentiation of stem cells through the direct detection of Cy5-tagged DNA. It was also reported that the accuracy and sensitivity of the system can be tuned by the degree of oxidation of graphene [143]. In a similar attempt, the substrate was used for measuring a wide range of dopamine concentrations (10^–4^ to 10^–9^ M) as well [144].

### 4.2. Nitrides-Supported SERS Substrates

**Figure 4:**
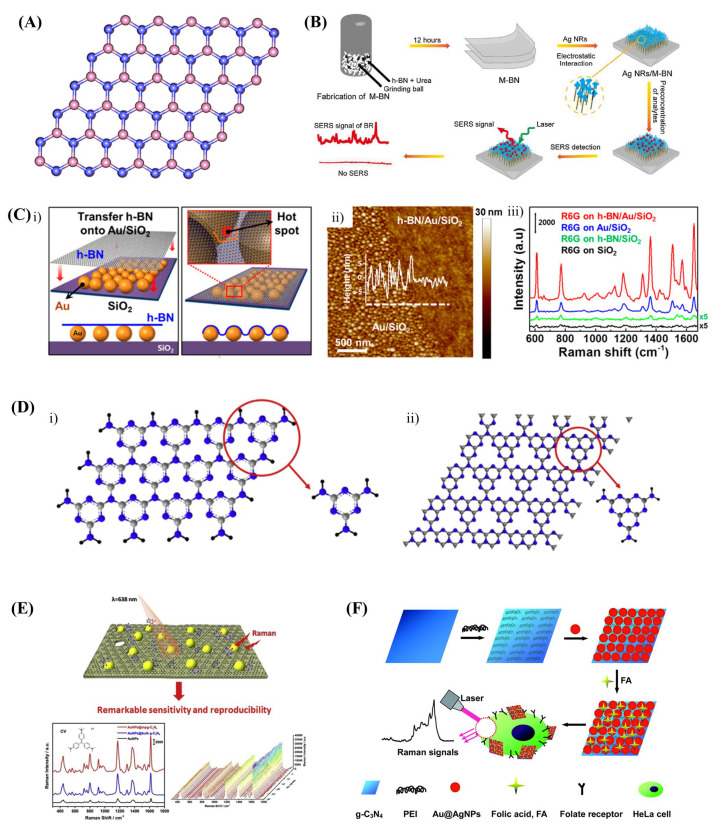
(**A**) Structure of 2D h-BN layer [152]. (**B**) Schematic diagram of fabrication of AgNRs/h-BN hybrid SERS substrate for the detection of bilirubin [74]. (**C**) (**i**) Schematic illustration, (**ii**) AFM image of h-BN layer on AuNPs and (**iii**) SERS spectra of Rh6G on different substrates [151]. (**D**) 2D layered (**i**) s-triazine and (**ii**) heptazine structures of g-C_3_N_4_ [153]. (**E**) AuNPs on the mesoporous g-C_3_N_4_ SERS substrate for the detection of 6-thioguanine [154]. (**F**) Schematic illustration of the fabrication of g-C_3_N_4_/Au@AgNPs hybrid as a SERS probe and its application in cancer diagnostics [155].

In plasmonic SERS substrates, the exceptional field enhancement is realized by the localization of SPR and their coupling. The 2D h-BN is an electrical insulator with a band gap of 6 eV [76]. It can be used for creating a nano-gap to prevent electron-transfer between nanoscale plasmonic surfaces and facilitate the localization of surface plasmon resonance [145]. The shielding property of h-BN has already been demonstrated for field-effect transistors [146,147]. The composite of 2D h-BN (Figure 4A) and plasmonic nanostructures can combine electromagnetic enhancement with dipole-coupled chemical enhancement of the h-BN [148]. The atomically thin h-BN nanosheets, wrapped over plasmonic nanoparticles, can concentrate analytes on the surface and enhance the Raman signal by a further two orders [149,150]. A detailed experimental and theoretical mechanistic study revealed that the nano-gap between the plasmonic NPs by the h-BN spacers (Figure 4C) facilitates a stronger electromagnetic field and thus higher signal enhancement [151]. In general, h-BN-wrapped plasmonic NPs SERS substrates show excellent stability and reproducibility.

Yang et al. have developed SERS active boron nitride nanosheet/AgNPs hybrids to investigate the impact of urea, uric acid, and creatinine on the steric configuration of bovine haemoglobin [123]. Similarly, a recyclable SERS substrate was fabricated by laminating the silver nanoarrays using 2D BN nanosheets for label-free detection of bilirubin in complex biological samples with higher sensitivity and durability [74]. Eventually, the substrate was able to detect bilirubin in blood with LOD as low as 2.5 × 10^−8^ M prompted by the higher affinity for hydrophobic bilirubin molecules with a BN surface (Figure 4B).

Graphitic carbon nitride (Figure 4D) is another important layered nitride that has been recognized widely as a support for nanoparticle dispersion. Wang et al. have synthesized AuNPs on the mesoporous g-C_3_N_4_ (Figure 4E) and achieved a wide linear range from 6 × 10^−7^ to 4.8 × 10^−5^ M and LOD of 2.4 × 10^−7^ M with a 6-thioguanine molecule [154]. Thermal annealing of g-C_3_N_4_/AuNPs at 350 °C improved the SERS signal further and enables the detection limit of uric acids at an ultralow level of 10^−11^ M [156]. With the help of g-C_3_N_4_ nanosheet/Au@AgNPs hybrid SERS substrates, folic acid detection down to 2.41 nM has been achieved (Figure 4F) [155]. Hybrids of h-BN and g-C_3_N_4_ have also been utilised as a support for AuNPs SERS substrates [154].

### 4.3. Black Phosphorous-Supported SERS Substrates

**Figure 5:**
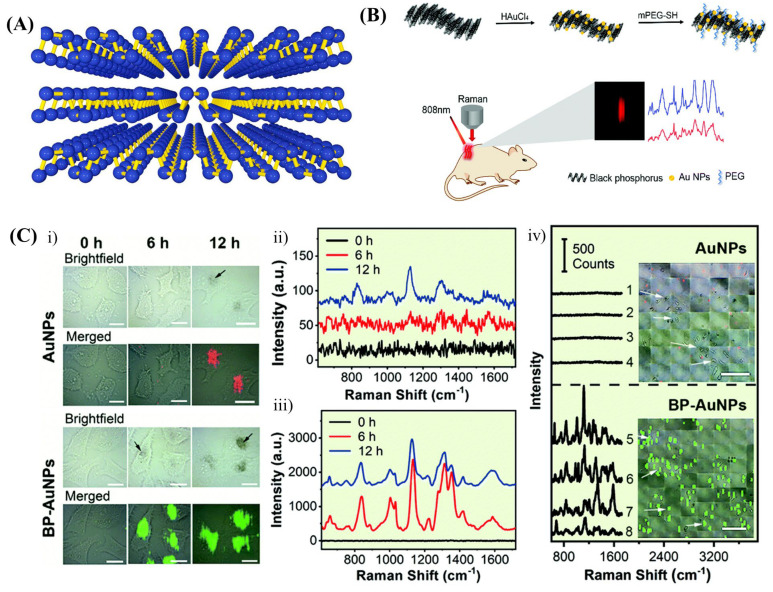
(**A**) Layered structure of Black Phosphorous [158]. (**B**) Schematic illustration of fabrication of BP–AuNPs SERS substrate and its application in monitoring cancer photothermal therapy [157]. (**C**) Fingerprint analysis and label–free NIR SERS imaging of living cancer cells: (**i**,**iv**) SERS imaging of Hep G2 cells (scale bar: 20 μm) (**ii**,**iii**) are the corresponding SERS spectra of Hep G2 cells induced by AuNPs and BP–AuNPs, respectively [124].

As discussed in Section 2.3, BP has unique optical properties, including a layer-dependent band gap and an anisotropic structure (Figure 5A), which is again advantageous when used as a nanoparticle support. For instance, Yang et al. have dispersed AuNPs on BP nanosheets to track the photothermal therapy effect [157]. The nanocomposite exhibited strong SERS signal enhancement as a combined effect of the EM and CM from AuNPs and BP nanosheets. Real-time SERS monitoring of the in vivo cancer photothermal therapy effect has been demonstrated with BP–AuNP-based SERS substrate (Figure 5B) [157].

Liu et al. [124] developed a BP nanosheets SERS probe to directly explore the fingerprint information of cancer cells (Figure 5C). Here, three model tumour cells, namely, human cervical carcinoma (HeLa) cells, mouse mammary cancer (4T1) cells and Hep G2 cells, have been taken for the study. First, the vibrational fingerprints of intracellular proteins of these living cells were distinguished. Then, the lateral mapping of the corresponding characteristic peak was obtained label-free. Although there are some overlaps between the 4T1, HeLa and Hep G2 cell groups, they can be discriminated by Principal Component Analysis–Linear Discriminant Analysis using scatter plots of the first and second discriminant functions.

### 4.4. MXene-Supported SERS Substrates

**Figure 6:**
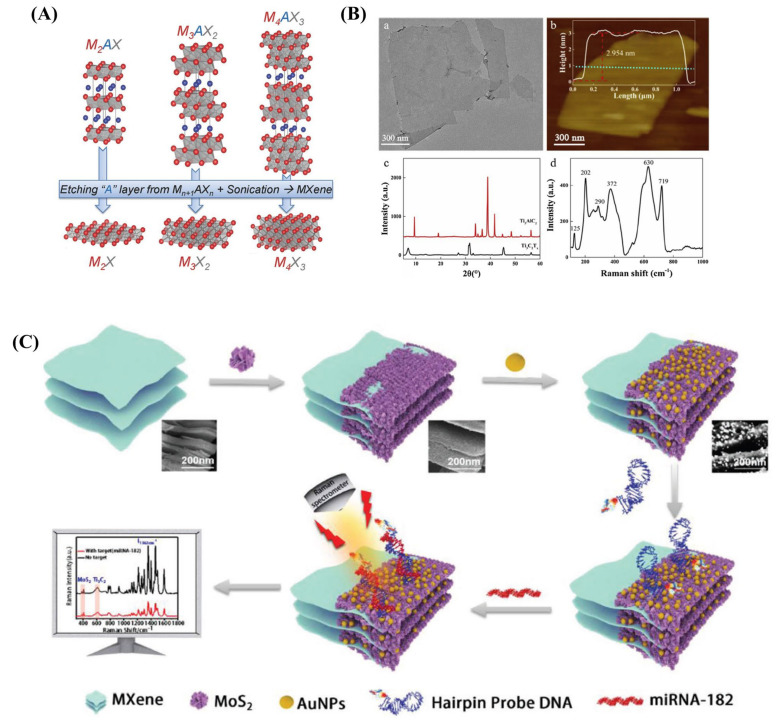
(**A**) Structure of MAX phases and the corresponding MXenes [161]. (**B**) (**a**) TEM, (**b**) AFM, (**c**) XRD and (**d**) Raman spectra of Ti_3_C_2_Tx nanosheets [159]. (**C**) Schematic illustration of a self-calibrated SERS strategy based on Mxene/MoS_2_@AuNPs ternary system for the ultrasensitive detection of cancer-related miRNA–182 [126].

MXenes have both EM and CM Raman signal enhancement, inherently [90]. In addition, their 2D structure (Figure 6A) with high surface area (typically, around 100 m^2^/g after delamination) makes them a suitable substrate for nanoparticle dispersion. For instance, Ti_3_C_2_Tx MXene was used as a substrate for dispersing AgNPs that turned into a sensitive, stable and uniform SERS substrate for detection of dopamine and adenine molecules at concentrations as low as 10^−8^ M (Figure 6B) [159]. The peak intensity was a logarithmic function of concentration with a linear response range from 5 × 10^−6^ to 5 × 10^−8^ M. The substrate demonstrated good detection capabilities in both serum and DI water samples.

Being a 2D nanostructure, MXenes easily form stacks with other 2DNS, such as TMD nanosheets. Liu et al. developed a novel synergistic self-calibrated SERS strategy using a 2D–2D stack (Figure 6C), MXene/MoS_2_, as a support for AuNPs dispersion for the ultrasensitive detection of cancer-related miRNA-182 [126]. Here, the substrate is self-calibrated using the 382 and 402 cm^−1^ modes of MoS_2_ and the 611 cm^−1^ mode of MXene. Another unique 2D–2D hybrid was developed by anchoring vertically aligned MoS_2_ nanosheets on MXene. The dispersed AuNPs formed an average nano-gap of 2.2 nm on this hybrid. It offered uniformly distributed hotspots, leading to the maximum SERS signal amplification with hairpin probe DNA, tagged with Cy5. For miRNA-182, the linear detection window was as wide as 10 aM to 1 nM with an ultralow detection limit of 6.61 aM.

The multifunctional capability of Ti_3_C_2_Tx MXene was demonstrated by Wei et al. with a dual-mode ECL/SERS immunoassay for the ultrasensitive detection of a harmful bacterium, Vibrio vulnificus [160]. Using the R6G-tagged hybrid, the bacterium could be detected with a linear range and limit of quantification (LOQ) from 10^2^ to 10^8^ CFU/ mL and 10^2^ CFU/mL, respectively. The complementary dual-mode signal helps mutual verification and ensures accuracy and reliability.

A typical sandwich immunosensor was developed by dispersing Fe_3_O_4_@AuNPs on Ti_3_C_2_Tx where the target analyte is selectively captured followed by magnetic pre-concentration. In this immunoassay, Medetalibeyoglu et al. used a 4-mercaptobenzoic acid-labelled MoS_2_ nanostructure as support for AuNPs, a reporter probe for CEA detection [127]. Similarly, an aptasensor was developed by Zheng et al. for the quantitative detection of ochratoxin A (OTA) with internal standard techniques [128]. Here, Au-Ag nanoparticles were conjugated with OTA aptamers and dispersed on MXene nanosheets (Figure 4B). Upon the addition of OTA, an aptamer/OTA complex is formed, which causes Au-Ag nanoparticles to separate from MXene nanosheets. Eventually, the Raman signal forms OTA changes with respect to that of MXene nanosheets, the internal standard.

### 4.5. Transition Metal Dichalcogenide-Supported SERS Substrates

The development of metal nanoparticles decorated 2DNS for SERS opens up possibilities for the analysis of microbes. The AuNPs dispersed HfTe_2_ SERS substrate with an EF of 1.7 × 10^8^ could detect, discriminate and quantify four prevalent food-borne pathogenic bacteria [111]. The LOD for the detection of E. coli, S. aureus, Salmonella and Listeria was 10 CFU/mL, commonly. By combining this capability of discrimination with the PCA algorithm, it was possible to categorize bacteria [46].

**Figure 7:**
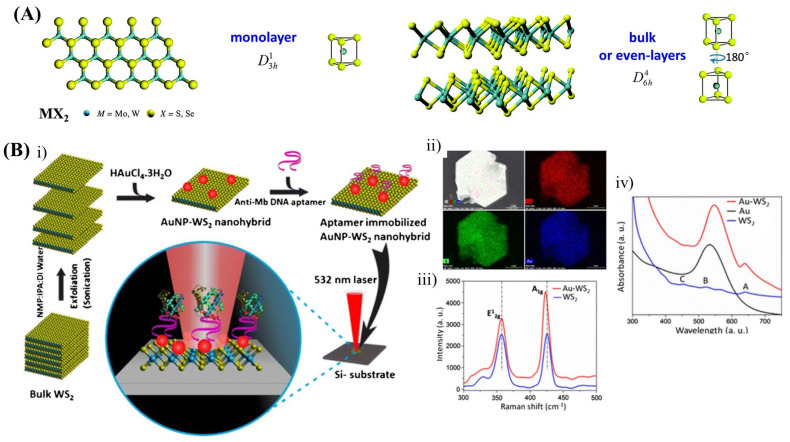
(**A**) The lattice structure of bulk and monolayer TMDs [162]. (**B**) (**i**) Schematic illustration of stepwise fabrication of Au−WS_2_ nanohybrid-based SERS substrate for the detection of myoglobin, (**ii**) EDX elemental mapping, (**iii**) Raman spectra and (**iv**) UV–Vis spectra of the nanohybrid [131].

Tungsten disulphide 2D heterostructure was used for label-free SERS aptasensors where AuNP-decorated WS_2_ nanosheets were used for the detection of myoglobin (Figure 7B) [131]. Similarly, AuNP-decorated MoS_2_ nanoflowers were used for the detection of free bilirubin in human blood. The 9-order signal enhancement helped sensing bilirubin molecules at levels ranging from pM to hyperbilirubinemia [130]. Yuan et al. developed AuNPs-modified MoS_2_ nanosheets for the detection of dopamine [47]. Here, dopamine is self-polymerized and aggregates AuNPs with an average 2 nm nano-gap. Subsequently, the hybrid is attached to the MoS_2_ nanostructure resulting in a 7-order SERS signal enhancement.

### 4.6. 2D MOF/COF-Supported SERS Substrates

Owing to an exceptionally high surface area, as high as 1200 m^2^/g, inherently abundant anchoring sites and tuneable band structure, 2D MOFs and COFs could be a suitable support for the dispersion of nanoparticles. Hybrid 2D MOF/COF-supported plasmonic nanoparticle SERS substrates have been developed for biomolecule sensing towards food safety monitoring and environmental analysis. Polycyclic aromatic hydrocarbons were detected in the range of 0.1–50 μM by employing COF/Au nanocomposites as SERS substrates [134]. Lai et al. developed an in situ method to grow hybrid core-shell Au@Ag NPs onto 2D Ni-MOF nanosheets, which were used as SERS substrates for the detection of synthetic pesticides and herbicides [133].

## 5. A Comparative Statement

The examples so far clearly indicate the potential of 2DNS as a SERS substrate for biomolecule sensing. Among them, graphene and its derivatives have been extensively used in SERS substrates as a support. The easy and cost-effective synthesis of graphene from graphite is the primary reason for its popularity. Even though they can be good at anchoring plasmonic nanostructures, their direct contribution to the signal enhancement is marginal (up to 10^2^ times). The biocompatibility of graphene and its changes with functionalization have been widely studied [163,164,165]. TMDs are the next popular 2DNS in SERS substrates. However, their biocompatibility has to be studied further under physiological conditions [166,167]. Easy, scalable synthesis routes, such as GO exfoliation, are needed to make TMD-based SERS substrates affordable. Despite the rich distribution of polar bonds in nitride 2DNS that help dipole-coupled signal enhancement, they have not been greatly explored as SERS substrates, particularly for biomolecule sensing. Oxides generally have a wider band gap, which requires higher energy for excitation. Most biomolecules undergo degradation under high-energy radiation. However, the band gap of these layered oxides, hydroxides and double hydroxides can be reduced considerably by doping and defect enrichment. MXenes and BP nanosheets are a relatively newer class of 2DNS for SERS applications. The highly conductive, metal-rich MXene 2DNS may have enormous unexplored potential as it can contribute both EM and CM in addition to anchoring nanoparticles. Despite having added advantages, MXenes are facing challenges due to their high cost and poor environmental stability. Similarly, stability is the bigger bottleneck for the MOFs and COFs to be deployed in SERS substrates, particularly for biomolecule sensing. While the technology is maturing, the cost factor, biocompatibility and stability at physiological conditions need to be addressed. Table 3 summarizes the different 2DNS SERS substrates, their enhancement mechanism, average enhancement factor and related advantages and disadvantages.

## 6. Current Technological Challenges and Opportunities

As discussed so far, 2DNS have comprehensive advantages both as a standalone signal enhancer and as support for the dispersion of plasmonic nanostructures. However, there are challenges and associated opportunities in practical applications.

In general, the SERS enhancement factor from 2DNS is substantially low compared to plasmonic substrates since they have CM contribution only. However, its ability to accumulate analytes through various weak interactions can address this limitation to a certain extent. Additionally, suitable functional groups need to be identified and associated methods for functionalization need to be developed.

Metal-rich 2DNS, such as MXenes, can offer a competitive signal enhancement. Due to quantum confinement, some of these 2DNS exhibit strong fluorescence when they are scaled down to a 2D structure. To address this problem, suitable fluorescence quenching methods need to be developed. On the one hand, MXenes and BP nanosheets have critical issues with chemical and environmental stability that leads to poor structure and function preservation, which restricts their practical applicability. Therefore, MXenes with water and air stability should be developed. On the other hand, graphene, TMD and nitride 2DNS are relatively cost-effective. Other 2DNS, particularly MXenes and BP nanosheets, are costly mainly due to their tedious synthesis procedure and, to a certain extent, raw material cost. While pushing the boundaries of signal enhancement by 2DNS, the cost factor also has to be considered.

Systematic investigation of biocompatibility is also required. In addition, developing novel scalable methods for surface functionalization to make these 2DNS highly water dispersible, biocompatible and biodistributable. Prior to any clinical translation, it is crucial to properly evaluate the biosafety and toxicity of these 2D nanostructures. Generally, surface heterogeneity is the major hurdle in obtaining reliable results from SERS-based sensors, particularly when EM is dominant. With their 2D structures, these layered materials offer an opportunity to improve homogeneity.

Another important extrapolation of 2DNS SERS is in food quality monitoring. It allows the easy detection of contaminants, such as adulterants and preservatives, and pesticides. However, the toxicity of these SERS substrates has to be deeply studied. Technologies for the safe use of these substrates with edibles need to be developed.

## 7. Conclusions and Future Perspectives

A thorough assessment of the most recent studies on 2DNS-based SERS substrates, such as graphene, h-BN, g-C_3_N_4_, TMDs, BP, MXenes, oxides and their heterostructures, for biomolecule sensing has been presented. Their role as a direct Raman signal-enhancing platform and support for plasmonic nanoparticles has been separately reviewed. 2DNS have seen impressive progress and have significant prospects in SERS applications. In terms of cost and environmental stability, graphene, TMDs and nitrides have been identified as suitable substrates. On the other hand, MXenes have shown good potential due to both EM and CM contributions, despite stability issues and there not being many attempts with this relatively younger material. The defect-induced, dipole-coupled SERS signal enhancement in oxides and nitrides needs to be explored further.

The major challenges associated with these 2DNS are their cost, stability and biocompatibility. Cost-effective scalable synthesis methods are required for the preparation of new 2DNS, such as BP and MXenes. The surface homogeneity, and thus the reliability and reproducibility of the signal, can be improved using 2DNS substrates. SERS-based biomolecule sensing is rapid, non-destructive and cost-effective compared to conventional methods. The progress in 2DNS SERS substrates clearly indicates that they can be potential future substrates with further technological advancements. The simple detection principle is broadly applicable to various analytes, including pathogens, biomarkers, drugs and food adulterants. With technological advancements, it is expected that 2DNS will eventually become a commercially viable SERS substrate for biomolecule sensing.

## Figures and Tables

**Table 1:** Representative examples of 2DNS-SERS substrates (Rhodamine 6G (Rh6G); Rhodamine B (RhB); Malachite Green (MG); Methylene Blue (MB); Crystal Violet (CV).

2DNS-SERS Substrate	Probe Molecules	Mechanism	EF	Ref.
Graphene				
Graphene	Rh6G	CM	1.7 to 5.6	[32]
UV/Ozone-GO	RhB, Rh6G, and CV	CM	∼10^4^	[33]
rGO	Rh6G	CM	∼10^3^	[34]
AgNPs/rGO	Rh6G	CM + EM	2.3 × 10^8^	[35]
AuNPs/GO/CW	Rh6G	CM + EM	1.0 × 10^6^	[36]
AgNPs/rGO	RhB	CM + EM	2.0 × 10^7^	[37]
AuNPs/rGO/	MG	CM + EM	3.8 × 10^3^	[38]
AgNPs/CVD Graphene	Rh6G	CM + EM	∼10^9^	[39]
TMD				
TiS_2_	Rh6G	CM	3.2 × 10^5^	[40]
1T-W(MoTe_2_)	Rh6G	CM	1.8 × 10^9^	[41]
2H-TaS_2_	Rh6G	CM	1.3 × 10^14^	[42]
Oxygen incorporated MoS_2_	Rh6G	CM	1.4 × 10^5^	[43]
MoTe_2_	β-sitosterol	CM	1.3 × 10^4^	[44]
HfTe_2_	Rh6G, CV, MB, and MG	CM	∼10^6^	[45]
AuNPs/HfTe_2_	MB	CM + EM	1.7 × 10^8^	[46]
AuNWs/MoS_2_	Rh6G and MB	CM + EM	∼10^7^	[47]
Black phosphorous (BP)				
BPQDs/AgNPs/TiO_2_	4-MBA	CM + EM	2.5 × 10^5^	[48]
BP flakes	RhB	CM	∼10^6^	[49]
BP Nanosheets	Rh6G	CM	6.7 × 10^7^	[50]
AgNPs/BP	Interleukin-3 (IL-3) and procalcitonin (PCT)	CM + EM	∼10^14^	[51]
Nitride				
Hexagonal Boron Nitride (h-BN)	MG, MB and Rh6G	CM	∼10^4^	[52]
Fluorinated h-BN	Rh6G and CV	CM	∼10^8^	[53]
AgNPs/g-C_3_N_4_	CV	CM + EM	2.1 × 10^9^	[54]
Hydrophilic hydrophobic g-C_3_N_4_@Ag	MG	CM + EM	3.2 × 10^6^	[55]
AuNPs/g-C_3_N_4_	Rh6G and Melamine	CM + EM	∼10^8^	[56]
MXenes				
AuNPs/Mo_2_C MXene	MB	CM + EM	2.2 × 10^4^	[57]
Ti_2_N MXene	Rh6G	CM	∼10^12^	[58]
Ti_3_C_2_	MB	CM	∼10^5^	[59]
Ti_3_C_2_ MXene	MB	CM	2.9 × 10^6^	[60]
V_4_C_3_ and V_2_C	Rh6G	CM	∼10^5^	[61]
AuNPs/TiC	Chlorpromazine	CM + EM	∼10^9^	[62]
TiVC	Rh6G	CM	3.3 × 10^12^	[63]
Nb_2_C, Mo_2_C, Ti_2_C, V_2_C, Ti_3_C_2_, Mo_2_TiC_2_, and Ti_3_CN	Rh6G	CM	-	[64]
2D MOFs/COFs				
Co-MOFs	Rh6G	CM	-	[65]
AuNPs/COF-paper	PAHs	CM + EM	12 to 194	[66]

**Table 2:** Comparison of various 2DNS-supported plasmonic nanoparticle SERS substrate used in the detection of biomolecules.

2DNS Support	Nanoparticle	Sample	Target	LOD	Ref.
Graphene-supported SERS substrates					
Graphene	AuNPs	DNA oligonucleotides	DNA	10 × 10^−12^ M	[115]
GO	popcorn-shaped AuNPs	Culture Collection	HIV DNA and bacteria	10 CFU/mL	[116]
GO	AuNPs	DNA sequence	DNA	10 × 10^−15^ M	[117]
GO	AuNPs	muscle tissue of the MBT and pork samples	DNA	10^−14^ M	[118]
GO (encapsulated)	AuNPs	saliva	MERS-CoV	0.525 pg/mL	[119]
GO	Au nanorods	serum	hepatitis B surface antigen	0.05 pg/mL	[120]
GO	AuNPs	serum	prostate-specific antigen	0.23 pg/mL	[121]
GO	AuNPs	Hep-G2 liver cancer cells	doxorubicin	-	[122]
Nitride-supported SERS substrates					
BN	Ag nanoarrays	blood	bilirubin	2.5 × 10^−8^ M	[74]
BN	AgNPs	bovine haemoglobin	urea, uric acid and creatinine	-	[123]
Black Phosphorous-supported SERS substrates					
multi-layer BP	AgNPs	Human lung carcinoma	Exosome	-	[50]
BP	AuNPs	Hep-G2 live cell	Hep-G2 cells	-	[124]
BP	AgNPs	Serum	LPS, IL-3, and PCT	10^−9^ M, 10^−12^ M and 10^−13^ M	[51]
MXenes-supported SERS substrates					
MXene	Ag nanorods	soil	PCB-77 and PCB-3	2 × 10^−10^ M and 2 × 10^−9^ M	[125]
MXene/MoS_2_	AuNPs	human serum	miRNA-182	6.6 × 10^−10^ M	[126]
Ti_3_C_2_Tx MXene	AuNPs	serum	adenine	10^−8^ M	[87]
MXene/MoS_2_	AuNPs	Bovine serum albumin	carcinoembryonic antigen	0.033 pg/mL	[127]
Nb_2_C and Ta_2_C MXenes			SARS-CoV-2	5 × 10^−9^ M,	[112]
MXenes	Au−Ag NPs	bovine serum albumin	Ochratoxin A	1.3 × 10^−12^ M	[128]
Ti_2_C MXene	Au–Ag NPs	food	carbendazim	0.01 × 10^−6^ M	[129]
TMD-supported SERS substrates					
MoS_2_	-	serum	CA19-9	3.4 × 10^−4^ IU/mL	[111]
MoS_2_	AuNPs	serum	bilirubin	10^−12^ M	[130]
MoTe_2_	Ag nanorods	Phosphate buffered saline	β-sitosterol	10^−9^ M	[44]
HfTe_2_	-	-	uric acid	0.1 × 10^−6^ M	[45]
HfTe_2_	AuNPs	-	foodborne pathogenic bacteria	10 CFU/mL	[46]
WS_2_	AuNPs	serum	cardiac marker myoglobin	0.5 × 10^−18^ M	[131]
2D MOF/COF-supported SERS substrates					
Cu-TCPP(Fe)	AuNPs	Saliva	Glucose	3.9 × 10^−6^ M	[132]
Ni-MOF	Au@AgNPs	-	thiram, diquat, and paraquat	87.1, 188.8, and 8.9 μg/L	[133]
COFs	AuNPs	-	PAHs	-	[134]

**Table 3:** Overview of different 2D nanomaterial SERS substrates.

2DNS	Common Preparation Method	Enhancement Mechanism	Typical EF	Advantages	Disadvantages
Graphene, GO	Exfoliation, CVD	CM	≤10^3^	Easy preparation, lower cost, and biocompatibility.	Low EF
Nitride (h-BN, g-C_3_N_4_)	Exfoliation, CVD	CM	≤10^4^	Thermal conductivity, mechanical, chemical and thermal stability	Low EF
BP	Exfoliation	CM	≤10^6^	Higher surface-to-volume ratio, anisotropy, low toxicity	Tendency of oxidation
MXenes	Chemical etching delamination	CM + EM	≥10^6^	Highest enhancement for a 2DNS, low toxicity	Tendency of oxidation, harsh preparation conditions
TMD	Exfoliation, CVD	CM	≤10^6^	Tunable bandgap, layer-dependent behaviour, high stability	Phase transition decreasing EF
2D MOF/COF	Chemical synthesis	CM	≤10^6^	large specific surface area, easy customization, biocompatibility	Poor stability

## Data Availability

Not applicable.

## Abbreviations

2DNS	Two-dimensional nanostructures
AgNPs	Silver Nanoparticles
AlN	Aluminium Nitride
AuNPs	Gold Nanoparticles
BP	Black Phosphorus
CFU	Colony-Forming unit
CM	Chemical Mechanism
COFs	Covalent Organic Frameworks
CT	Charge Transfer
CV	Crystal Violet
CVD	Chemical Vapour Deposition
DNA	Deoxyribonucleic acid
ECL	Electrochemiluminescence
EF	Enhancement Factor
EM	Electromagnetic Mechanism
g-C_3_N_4_	graphitic carbon nitride
GERS	Graphene Enhanced Raman Scattering
GO	Graphene Oxide
h-BN	hexagonal Boron Nitride
HIV	Human immunodeficiency virus
HOMO	Highest Occupied Molecular Orbital
LOD	Limit of Detection
LOQ	Limit of Quantification
LSPR	Localized Surface Plasmon Resonance
LUMO	Lowest unoccupied Molecular Orbital
MB	Methylene Blue
MG	Malachite Green
MOFs	Metal Oxide Frameworks
MoOx	Molybdenum Oxide
MoS_2_	Molybdenum Sulphate
NPs	Nanoparticles
NWs	Nanowires
OTA	Ochratoxin
PCA	Principal Component Analysis
rGO	reduced Graphene Oxide
Rh6G	Rhodamine 6G
RhB	Rhodamine B
SERS	Surface-Enhanced Raman Scattering
TaN	Tantalum Nitride
TiAlN	Titanium Aluminium Nitride
TiN	Titanium Nitride
TiO_2_	Titanium Oxide
TMDs	Transition-metal dichalcogenides
WN	Tungsten Nitride
WOx	Tungsten Oxide
WS_2_	Tungsten Sulphate
